# Occupational Stress: A Concept Analysis with Implications for Immigrant Workers’ Mental Health in the United States

**DOI:** 10.1155/2023/1332479

**Published:** 2023-04-13

**Authors:** Reimund Serafica, Timothy Grigsby, Bradley Donahue, Lorraine Evangelista

**Affiliations:** 1 School of Nursing, University of Nevada, Las Vegas, Nevada, USA; 2 School of Public Health, University of Nevada, Las Vegas, Nevada, USA; 3 Department of Psychology, University of Nevada, Las Vegas, Nevada, USA

## Abstract

**Background.:**

Occupational stress is a phenomenon affecting people worldwide. Investigating occupational stress among immigrant worker populations will unravel some of the intricacies of this condition and its psychological effects on this population.

**Aim.:**

This paper conceptually examined occupational stress within the context of immigrant workers’ mental health and offer an operational definition to aid nurse researchers, educators, and practitioners in assessing and managing patients and developing culturally appropriate interventions for this population.

**Design.:**

Walker and Avant’s eight-step concept analysis is used as an organizing framework.

**Data Source.:**

MEDLINE, CINAHL, OVID, PubMed, and APA Psych Info.

**Review Methods.:**

Keywords job stress, immigrant work stress, occupational stress scale, immigrant work-related stress, and mental health were used. The search yielded 142 articles; 17 were selected based on the effect of work stress on mental health.

**Results.:**

This analysis found that occupational stress can be attributed to communication problems, alienation, discrimination, and barriers to work-life balance can cause negative consequences among immigrants. An operational definition is also provided.

**Conclusion.:**

There is a growing need to examine closely and differentiate between occupational and acculturative stress to navigate a more profound understanding of how these conditions negatively complement each other.

## 1. Introduction

Millions immigrate annually and must adapt to new cultures and conditions [1, 2]; however, these events may induce acculturative stress [3], which can drain mental resources and induce psychological conditions like anxiety, depression, cognitive impairment, and mood disorders [4]. Stress is a significant predictor of poor physical and mental health [5] and is correlated with increased anxiety and depressive symptoms. Depression can impair judgment, risk perceptions, concentration, and concern for personal safety [5], as evidenced in a recent study that links depression to substantial increases in occupational injury [6]. Research also demonstrates that people who feel stressed or depressed are more likely to engage in negative coping behaviors (e.g., substance abuse), increasing personal risks [5, 7]. Psychosocial factors that relate to the immigrant experience, such as low income, family separation, limited social support, marginalization, and an unhealthy lifestyle [7] may exacerbate these issues.

While studies of immigrant populations focus on workplace hazards, few consider elements affecting overall health. From a cost-benefit standpoint, it is critical to understand the compounding factors affecting occupational stress among immigrant populations to prevent costly, adverse health outcomes and, most importantly, improve their quality of life. As a secondary consideration, organizations can also benefit from such investigations as the indirect costs of occupational stress, such as absenteeism and low productivity, affect an organization’s financial health [8].

Nurses and other health professionals are uniquely positioned to improve the physiological and psychological well-being of immigrants [4] as they are equipped to assess, intervene, and offer remedial occupational stress-related psychological dysregulations to prevent mental disorders.

## 2. Concept Selection and Methodology

While occupational stress is a recognized issue [6, 9–11], it is rarely examined in conjunction with the mental health of immigrant workers. Investigating the connection between occupational and acculturative stress may help capture the intricacies of institutionally and socioeconomically disadvantaged immigrants and the effects of these stressors on immigrant well-being.

For this concept analysis, we applied Walker and Avant method as an organizing framework due to its suitability [12]. The process involves the following eight steps:

1. Select a concept
2. Determine the purpose (aim) of the analysis
3. Identify all uses of the concept
4. Determine defining attributes of the concept
5. Describe the model case
6. Describe additional cases
7. Identify antecedents and consequences
8. Describe empirical referents

The model and additional cases (i.e., borderline and contrary cases) illustrate the impact of occupational stress on immigrant worker well-being.

A detailed literature review was performed in MEDLINE, CINAHL, OVID, APA Psych Info, and PubMed using the keywords *job stress, immigrant work stress, occupational stress scale*, and *immigrant work-related stress*. No limit was placed on the year of publication. The initial search returned 142 publications. The titles were screened with 101 articles eliminated. Following the abstract review, 17 articles published between 2002 and 2022 were finally selected for inclusion in this concept analysis of occupational stress. We limited our search to studies in the United States and Canada. The articles were selected based on the specificity (i.e., immigrants working in North America) and the effect of work stress on mental health. Three populations were dominant in the literature: Latinos, Filipinos, and Koreans.

## 3. Aims of the Analysis

This concept analysis examines and describes occupational stress within the context of immigrant workers’ mental health and to offer an operational definition of the term as it applies to mental health to aid nurses, researchers, educators, and practitioners in assessing patients and developing culturally appropriate interventions.

## 4. Uses of the Concept

Walker and Avant [12] recommend searching multiple sources, including dictionaries and the literature to support the identification of the concept. A review of the literature revealed several definitions of the term “occupational stress” although none specifically related to the context of mental health among immigrant workers. Several of the reviewed studies assessed occupational stress, but because the national origin and workplace samples were limited to specific countries, they did not thoroughly evaluate immigrant workplace acculturation. As a result, these studies cannot account for the diverse experiences of immigrant populations or their differing mental health outcomes.

The Online Webster Dictionary was searched for a definition of occupational stress. However, the word stress existed without the word occupation. Stress is as a state resulting from a stress especially one of bodily or, mental tension resulting from factors that tend to alter an existing equilibrium [13]. In the literature, occupational stress is not an acute or toxic condition that can be cured through treatment [6, 9, 14]. Rather, it is a chronic condition that requires an understanding of the epidemiology or life history of the problem prior to exploring cultural appropriate intervention alternatives [11].

We further classified subcategories of occupational stress to: sociocultural, psychological, and health-related issues based on the recent literature.

### 4.1. Sociocultural Context.

The sociocultural context surrounding immigrant workers is crucial, considering the conditions created by acculturation, internal and external to the work environment, can be detrimental to the immigrant’s well-being. In work centers, discrimination is a significant stressor for foreign-born workers, exemplified in nonstandard wages, limited choices, and abuse (e.g., Filipino migrant nurses facing bullying, alienation, and resettlement while integrating into the host country) [15]. Workers from less-developed countries also associate discrimination incited by the host country with depression [16]. In one study, Latino workers express the importance of management fairness and supervisor support for their well-being [17].

Occupational factors are only part of the problem; external factors also influence the mental health of immigrants. For Latinos, a population considered loyal and family-oriented [18], deficient familial support and frequent conflicts can detract from their ability to manage workplace demands, which is exacerbated by stressors associated with a lack of English language proficiency and legal status concerns. Due to the limited resources that immigrants may have to manage hardships at work, even low levels of support may strengthen the positive link between stressors and well-being [5, 19].

### 4.2. Psychological Context.

Various work-related events also affect the psychological well-being of immigrants. Occupational stress is associated with depression, anxiety, a need for recovery, and life satisfaction [20]. Job security is also connected to stress [17], whereas job demands, job control, and interpersonal conflict are associated with depression [9, 18]. Furthermore, social isolation and working conditions are risk factors associated with both anxiety and depressive symptoms [15]. An additional and unique element to the immigrant experience is the stress induced by legal status. A study of undocumented Latino day laborers indicates that many avoid public exposure, fearing arrest or deportation [21]. This stress can detrimentally impact the mental health of these workers, heightening their depression risk.

Similar findings show that US-based Filipino nurses face resettlement demands because of unexpected social and living environments; they also experience communication issues, discrimination, and alienation, which intensify work-related stressors [22]. Specific stressors highlighted by study participants included loneliness, self-reliance, work-life balance, and financial and economic issues [22]; work-specific stressors included a lack of support from nursing administration, insufficient time to complete tasks, leading without adequate experience, and unreasonable patient demands [22].

### 4.3. Health-Related Issues.

The occupational stress immigrant workers experience is multidimensional and intricate, complicating the identification of these stressors and how they intersect. Furthermore, the cumulative effects of elevated stress levels over time have increasingly been linked with *allostatic load* (AL): a summary measure of the physiological “wear and tear” the body experiences with repeated bouts of stress, intensified by unhealthy behaviors or conditions (e.g., smoking; social isolation) [11, 23]. When AL remains outside normal ranges for prolonged periods, it can cause comorbidity risks, such as cardiovascular disease, diabetes, autoimmune disorders, and death [11]. AL among immigrants and foreign-born workers is well-documented in the literature [14, 24].

## 5. Defining Attributes

This step in Walker and Avant [12] method identifies the characteristics of occupational stress among immigrant workers that repeatedly appear when the concept is described. While a gap exists in occupational stress literature regarding the applicability of this stress to cultural groups, the characteristics present in existing relevant literature are categorized as follows:

1. The immigrant experience concerns exchange between two cultural environments: country of origin and the United States [1, 4, 25].
2. Acculturative stress significantly contributes to occupational stress.
3. Occupational stressors can cause physical and psychological/mental health problems [5, 7, 10].
4. Mental health problems derived from occupational stress can predict an immigrant worker’s overall life satisfaction and quality [20–22].

## 6. Constructing Cases

The following constructed cases illustrate a model case and two additional cases: borderline, and contrary case.

### 6.1. The Model Case.

Lorna, a registered nurse, immigrated from the Philippines 5 years ago and became a med-surge nurse in one of the largest healthcare systems in the southern United States. Despite networking with other Filipinos, Lorna misses her family and friends in the Philippines. She admits she struggles to acclimate to the American condition: living alone, driving in a large metropolitan city, eating different food, and understanding and adapting to the local accent.

Lorna has faced challenges assimilating to the US healthcare system, where she floats on different shifts. As she has gained experience, she has been assigned as the charge nurse, responsible for supervising 12 nurses. This position stresses her since she rarely has time to eat or hydrate, and she dreads her shifts, which are 12 hours but sometimes extend to 14 hours due to high-need, complex patients, and difficult family members and coworkers. During her first few months in the United States, Lorna experienced microaggressions from other nurses and racism and bullying from patients. Since arriving in the United States, she has gained significant weight and admits not recognizing herself in the mirror. She also confesses to staying in bed on her days off, thinking about work.

Lorna’s work situation affects her quality of life, eating, focus, and sleep. Her provider referred her to a psychiatric mental health nurse practitioner, who diagnosed her with depression and anxiety. She was also diagnosed with insomnia due to changing shifts. While Lorna is a green card holder and can apply for US citizenship, she is uncertain if she wants to remain in the United States.

The above-given case adequately represents a model case because Lorna has demonstrated the defining attributes of stress she endures after immigrating to the United States (e.g., acculturative stress). She is experiencing physical and psychological distress affecting her overall quality of life.

### 6.2. The Borderline Case.

Jose immigrated to the United States from Mexico 20 years ago. He is the sole provider for his wife and two children, and he and his family are naturalized US citizens. When he first arrived in the United States, Joe worked in construction; however, he sustained back injuries that prevented him from performing labor-intensive work. After losing his job, he was diagnosed with depression.

Jose currently works as a front desk manager in one of the top casino resorts in Las Vegas. He can speak English and Spanish and has no issues communicating with clients. While Jose admits he had difficulty learning the hospitality industry, he has developed an appreciation for the work, having moved from housekeeper to bellboy before becoming a front desk manager. He has considered gaining more training to work in upper management. In addition to his hospitality work, Jose moonlights as an Uber driver, working 8-hour shifts twice weekly to supplement his income.

In conversations with coworkers, Jose acknowledges the stress of living in America due to the culture, expectations, and living costs. He desires to own a landscaping business but fears this job would not be enough to provide for his family, considering he would lose the benefits (e.g., medical care) offered by his current job.

A borderline case is when a case includes most or some attributes of occupational stress among immigrants. Although most defining attributes apply to Jose, his ability to adapt in the face of adversity is noticeable.

### 6.3. The Contrary Case.

Born and raised in the United States in an affluent Midwest neighborhood, Angelo is the son of Korean immigrants who own several restaurants and a dry-cleaning chain. Angelo attended a private high school, was enrolled in a private college, and worked as a part-time IT technician assistant at his university. However, he was introduced to illegal drugs and excessive alcohol in college and eventually dropped out due to poor academic performance. Despite his parents’ interventions, Angelo has been in and out of rehab and recently suffered a significant relapse. He also suffers from intermittent visual and auditory hallucinations because of his substance abuse history.

Walker and Avant [12] explain that a contrary case is an example without defining attributes. The contrary case presented as experienced by Angelo demonstrates something that is not regarded as the main concept. Though Angelo suffers from psychological issues, these were not caused by his employment and can be attributed to a lack of self-agency and inefficient self-efficacy.

## 7. Antecedents

Walker and Avant describe antecedents as events that must occur before the concept materializes. One primary antecedent of occupational stress in immigrant workers is a change in the work environment, which can contribute to coping behaviors and mental health issues [5, 6, 18, 26]. Areas for consideration include problems with employment or the work environment, job dissatisfaction, work safety, stressful work conditions, long hours, sexual harassment, and other discord with supervisors [10, 15, 22, 27]. Cultural differences and other aspects of hostile work environments, including microaggressions, bullying, communication problems, discrimination, alienation, and intensify work-related stressors [5, 11, 20, 21].

## 8. Consequences

Walker and Avant [12] describe consequences as events that occur because of the concept. In this study, mental health issues are consequences of adverse occupational health incidents. Stress may be caused by structural and situational factors, like discrimination, poverty, family separation, social isolation, low control in the work environment, and harsh working conditions [5, 6], the latter exacerbating adverse mental health outcomes [5].

The literature cites multiple examples of work-related consequences affecting immigrant workers. For instance, overqualified and overeducated workers who accepted entry-level jobs in the United States experienced more adverse mental health outcomes than peers, including depression, anxiety, and psychological distress [22, 27]. Numerous studies have shown that immigrant farmworkers experience high stress, anxiety, and depression [6]. Others indicate that Latino day laborers frequently report sadness, hopelessness, and desperation due to stressful life and work conditions, particularly work-related injuries [21, 26]. In addition, a study of Korean immigrants showed that microbusiness owners (MBOs) experience greater emotional demands, with increased depressive symptoms related to long work hours and language difficulties [10]. Although emotional demands can harm the psychological health of MBOs, the effectiveness of job satisfaction and security in compensating for such adverse health effects is greater among MBOs than employees [10]. Furthermore, while one study demonstrated that Filipino nurses have the resources and skills to care for their emotional well-being, the participants admitted that working long shifts made socializing in the new country challenging [15]. Some studies indicated that Filipino nurses also suffer from work-related stress, sleep problems, anxiety, and depression [28, 29]. To relieve the high stress of acculturation and workforce integration, immigrants may rely on harmful coping mechanisms, like heavy drinking and tobacco use [28].

## 9. Empirical Referents

Empirical referents are categories or classes of phenomena that demonstrate the occurrence of the concept and illustrate how the concept can be recognized. They (a) show how the concept exists and is measured in the real world and (b) demonstrate the unquestionable existence of the concept through observable, measurable, and verifiable means. Researchers have used several questionnaires to measure occupational stress in immigrants; however, it is crucial to extract items from these questionnaires to identify the cause of stress and related mental health outcomes. Examples of these measures are as follows:

- A recent study captured job-related stress using 6 items based on the hispanic stress inventory (HSI) immigrant version [6].
- The migrant stress inventory (MSI) is a scale designed initially for migrant farmworkers [30], which is also used to target work-related stress.
- The daily hassles scale used in Filipino and other Asian studies measures job stress using 10 occupational-oriented items [27]. Examples include problems connecting with the boss, concerns about job security, and dissatisfaction with fellow workers, current duties, and the job overall.
- The revised occupational stress inventory (OSI-R) measures occupational satisfaction, strain, and personal resources. A subscale on occupation stress was used in a study involving Filipino nurses in North America [15].

## 10. Discussion

**Figure 1:**
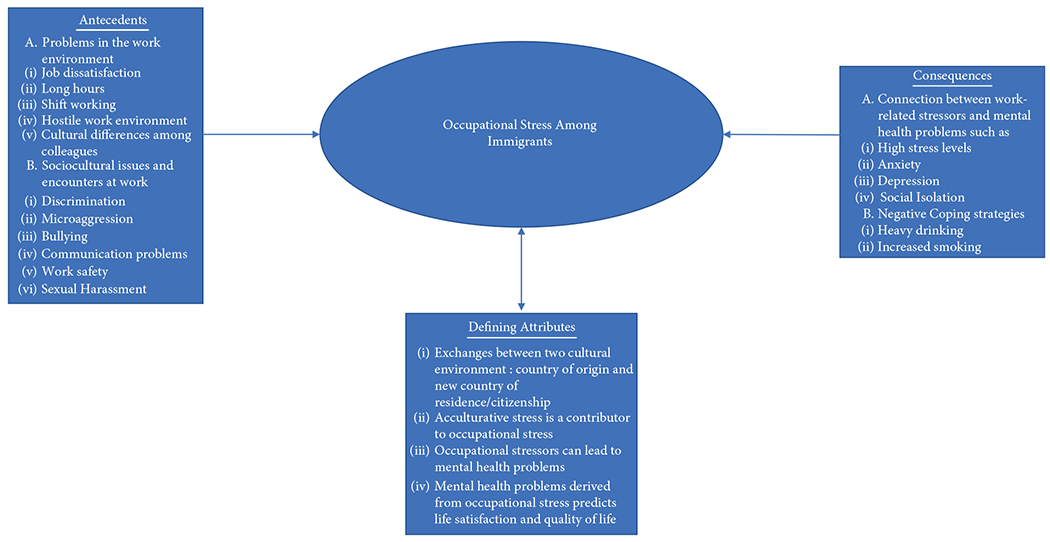
Representation of antecedents, consequences, and defining attributes to occupational stress among immigrant worker.

This paper conceptually and substantially examined occupational stress within immigrant workers’ mental health context, as depicted in Figure 1 and offers an operational definition of occupational stress. There is a connection between work-related occupational stress and mental health among working immigrants. In defining occupational stress as it relates to the mental health of immigrant workers, we offer this definition as a result of this concept analysis:

Occupational stress among immigrants is associated with stress-related factors brought cumulatively from places of employment after immigration that can cause higher negative emotions that lead to psychological distress to an individual and affect the overall quality of life.

Most immigrants come to North America to reunite with family or for financial or economic reasons. While immigrants in high-skill professional disciplines, such as nursing, are prone to occupational stress, daily stress can lead to job dissatisfaction, intent to leave, and mental distress (e.g., depression and anxiety). In addition, multifactorial occupational challenges such as communication, alienation, discrimination, and work-life balance can cause psychological consequences.

Injuries and below-average working conditions also result in different mental stress for Latino labor workers. Stress may be particularly problematic for immigrants, who may have fewer resources to manage it than their native-born counterparts. Furthermore, stress related to immigration status in a company with low social support and cultural barriers may exacerbate the adverse health effects of occupational stress for immigrant workers. Finally, understanding allostatic stress among immigrants may explain how adverse life and work conditions can contribute to future disease development.

The current evidence also raises important questions about discrimination and racism related to the immigrant experience. While racism, microaggressions, and bullying are not always consciously explicit or readily visible, these events can harm immigrants from an economic and mental health perspective. This concept analysis mirrors those of a previous study, which suggests that isolation, hatred, lack of respect, racism, and discrimination can lead to poorer health among people of color at all economic levels by exposing them chronically to unfair, race-based interpersonal treatment. These conditions can produce chronic stress among immigrants, contributing to job dissatisfaction and poor quality of life. This analysis also found that occupational stress may be attributed to harmful lifestyles such as poor nutrition, smoking, and substance abuse.

Instruments exist to measure occupational stress, but there is a growing need to (a) differentiate between occupational stress and acculturative stress and (b) cultivate a more profound understanding of how these stressors negatively contribute to the AL experienced by immigrants living and working outside their native country. Similar to one study, this concept showed that immigrant stress was influenced by situational (i.e., adapting to US work/living environments) and cultural contexts, contributing to the stress’s complexity and multidimensionality [28] and emphasizing the need to assess and manage occupational stress among these workers.

## 11. Implications for Nursing Research and Practice

North America admits hundreds of thousands of immigrants annually, and workforce diversity is emerging as one of the most pressing issues of organizational life. Thus, future research should employ new approaches to address gaps in our understanding of diversity and its implications for occupational stress and consider approaches to alleviate stressors in this population. This research should consider culturally congruent interventions (e.g., music therapy, tai chi, meditation, and yoga) to help immigrant workers manage occupational stress as well as culturally sensitive counseling, such as cognitive behavior coaching, to facilitate worker resilience. Mobile health, mindfulness apps, social media support groups, and other technological approaches can also aid in delivering culturally appropriate mental health interventions. Community-based participatory research (CBPR) is also suitable for assessing and evaluating immigrant health and safety, and exploratory studies can deepen investigation into immigrant and host-nation differences to identify and develop appropriate interventions and policies.

Nurses in practice play a crucial role in managing immigrant health. They must assess acculturation and occupational stress in patients, including their moods, anxiety levels, and suicidal thoughts; consider these factors’ influences on mental well-being (e.g., immigrant shift workers comprise a significant number of stress and sleep disorder cases); and refer patients reporting psychological distress for further assessment. Immigrants suffering from homesickness, loneliness, and isolation, in addition to work-related stress (e.g., discrimination, racism, and harsh working conditions), must be signposts for mental health referrals. Furthermore, nurses must encourage employers to practice empathy, consider immigrant employees’ cultural perspectives, and offer resiliency training to facilitate a healthy mental state for this population.

## Data Availability

The data supporting this concept analysis are from previously reported studies and datasets, which have been cited. The processed data are available from the corresponding author upon request.
